# Recurrent candida prosthetic endocarditis over fifteen years managed with medical therapy and four valvular surgeries: a case report and review of literature

**DOI:** 10.1186/s13019-015-0309-7

**Published:** 2015-07-30

**Authors:** Bishnu P. Dhakal, Curtis G. Tribble, James D. Bergin, Sean Winfrey, William H. Carter

**Affiliations:** 1 Department of Medicine, West Virginia University, Charleston Division, Charleston, WV USA; 2 Cardiothoracic Surgery, University of Virginia, Charlottesville, VA USA; 3 Cardiology Division, University of Virginia, Charlottesville, VA USA; 4 West Virginia School of Osteopathic Medicine, Charleston, WV USA; 5 Cardiology Division, West Virginia University, Charleston Division, Charleston, WV USA

**Keywords:** Candida, Prosthetic, Endocarditis

## Abstract

**Background:**

Candida prosthetic endocarditis (CPE) is an uncommon disease involving less than 1 % of infective endocarditis patients and associated with high recurrence rate. Immunosuppresion, intravenous drug abuse, cardiac surgery and indwelling foreign bodies are the major risk factors for CPE. There are very few reported cases of CPE where more than one surgery was performed and there has generally been limited follow up on these cases.

**Case presentation:**

We report a case of a 35 year old woman who had mitral valve annuloplasty complicated by recurrent episodes of CPE leading to multiple mitral valve replacements (MVR). She underwent MVR surgeries a total of four times over an eighteen year period and had good functionality during most of this time while being on antifungal suppressive treatment. This is a unique case in terms of numbers of surgeries performed, the length of the follow up and the involvement of three different Candida species.

**Conclusion:**

Current guidelines for the treatment of candida endocarditis recommend surgical treatment followed by long term antifungal therapy although the cure rate by all treatments is low. However we feel that based on this one case it is reasonable to consider multiple redo valve replacement surgeries in conjunction with antifungal treatment for selected patients stable enough to tolerate the surgery.

## Background

Candida prosthetic endocarditis (CPE) is an uncommon disease involving less than 1 % of infective endocarditis cases and up to 3.4 % of prosthetic valve infections. CPE has an incidence of less than 1 case per year even at tertiary care centers which is increasing secondary to intravenous drug abuse [1–4]. CPE is associated with a low cure rate with recurrence of up to 36 % and has 5-year survival of less than 50 % [1, 2, 4]. The high risk populations are those who are immunocompromised, intravenous drug abuser, previous cardiac surgery and those with indwelling foreign bodies such as catheters, pacemakers, or prosthetic joints [2, 5–10]. CPE usually leads to an acute and progressive valvular dysfunction causing multiple complications such as heart failure, embolic phenomena and stroke [1, 2, 11]. One possible explanation is the lack of blood supply to the prosthesis and surrounding tissue and subsequent impaired immune response. Furthermore candida can form a biofilm on the prosthesis surface which is resistant to treatment and thus may require lifelong suppressive therapy [2, 12]. Current guidelines recommend surgical treatment followed by long term antifungal therapy although the cure rate by all modality of treatments is low [13, 14]. Apparent long term cure have occurred by both medical and surgical therapy [2, 10, 13, 14].

## Case presentation

We report a case of a 35 year old female who had mitral valve (MV) commissurotomy and ring annuloplasty (32 mm Carperntier) on July 28, 1987 for rheumatic valvular disease. Fifteen months after this surgery, she presented with an embolic event to posterior cerebellar artery. Her blood cultures were positive for *Candida tropicalis*. Echocardiography showed moderate mitral stenosis with 0.5 × 0.4 cm density on the anterior portion of the ring behind the mitral valve consistent with fungal vegetation. She did not improve clinically after treatment with amphotericin B and required a 31 mm Medtronic Hall mechanical prosthesis on 10/17/88 which was followed by chronic outpatient suppressive treatment with fluconazole.

In August 1989, she presented to the hospital with pancytopenia, splenomegaly and renal failure. A large fungal vegetation was noted on the MV annulus and blood cultures were positive for a different species of candida (*C. parapsilosis*) leading to her third mitral valve procedure (second replacement) on 8/14/1989. She was discharged home and enjoyed a period of seven years with good functionality while being on 200 mg per day of fluconazole suppressive therapy.

In December 1996, she presented with three weeks of fever, marked splenomegaly and hemorrhagic infarct of the left optic disc. Her blood cultures grew *C. parapsilosis* prompting the initiation of amphotericin B and fluconazole therapy and surgical consultation at University of Virginia (UVa) for the third episode of CPE. The fourth mitral valve procedure (third replacement) was performed on 1/22/1997 at UVa followed by increased dose of fluconazole (400 mg per day).

In November 1998, she presented with continued fever, heart failure and overt pulmonary edema secondary to *C. tropicalis* prosthetic MV endocarditis with positive blood cultures. TEE showed dilated and hypokinetic right ventricle, severe tricuspid and mitral valve regurgitation with an LV ejection fraction of 35 %, and severely elevated pulmonary artery pressure (80–90 mmHg). The fifth, and last, mitral valve procedure [fourth replacement (St Jude Valve)] was performed on 11/23/1998 at UVa due to worsening heart failure and pulmonary edema. Due to recurrent CPE, she was also considered for heart transplant but repeat MVR along with ongoing antifungal treatment was thought to be better. She was discharged on liposomal amphotericin B three times a week to correspond with her visits for erythropoietin infusion secondary to pancytopenia before resuming fluconazole. She was closely followed on oral fluconazole at 800 mg daily from January 1999 until 2006. She consistently denied using any intravenous drug or medication noncompliance. During this 7 year period, she enjoyed an active lifestyle including camping and fishing.

Worsening pancytopenia and splenomegaly developed in the second half of 2006 secondary to recurrent *C. parapsilosis* MV vegetation with positive blood cultures which led to biventricular heart failure, multiorgan failure, pancytopenia and acute kidney injury requiring hemodialysis. The candida species was still sensitive to fluconazole, voriconazole, itraconazole, amphotericin B and 5-flucytosine. The patient was felt not to be a candidate for redo surgery and subsequently died in November 2006.

## Discussion

Our patient enjoyed a good quality of life for more than 18 years following her first valve surgery for CPE and 16 years following her first of multiple valve replacements for CPE. Moreover, she survived 8 years following her last of four mitral valve replacement surgeries on fluconazole suppressive therapy.

We were able to find only three other similar cases in the literature who had more than one surgery performed for CPE [1, 15, 16]. The first patient developed CPE three years after the valve replacement but had discontinued the suppressive antifungal therapy for 8 months before the recurrence [1]. Another patient was treated with caspofungin after aortic valve replacement (AVR) for CPE but developed recurrence two months later and required a second AVR with a cryopreserved homograft. The patient was then managed medically with liposomal amphotericin B followed by fluconazole [15]. The third patient was treated with amphotericin B for *C. parapsilosis* aortic valve CPE but developed recurrence after 4.5 months and died during reoperation [16]. The longest reported survival noted was for our patient who had two different types of candida species.

The common candida species causing endocarditis are *C. albicans* followed by *C. parapsilosis* [17]. The later organism now exceeds *C. albicans* in terms of frequency. Our patient, felt to be a non intravenous drug user, is unique in having two different candida organisms. API 20C [18] and Chromogenic medium [19] were used for isolation and appropriate identification of the candida species. Since these techniques are highly sensitive in identifying the type of candida species and it is very rare to have growth of different candida species during subsequent recurrences which makes us suspicious if the candida species was very slow growing and not detected during the microbiologic culture.

Infectious Diseases Society of America (IDSA) and European Society of Clinical Microbiology and Infectious Diseases (ESCMID) guidelines for treatment of native and prosthetic valve endocarditis recommend valve replacement along with antifungal treatment with amphotericin B with or without 5-flucytosine followed by long-term suppressive therapy with fluconazole [13, 14]. Immediate surgery has been assigned grade A recommendation by ESCMID and grade B by IDSA but both are level III evidence derived from opinions of respected authorities, based on clinical experience, descriptive case studies or expert committees. For those who are unable to undergo surgical removal of the valve, chronic lifelong suppression with fluconazole 400–800 mg (6–12 mg/kg) daily for lifelong is recommended [14]. Echinocandins may be more effective for candida species producing biofilms since a trend towards better outcome in patients receiving caspofungin at diagnosis has been reported [9].

Despite the guidelines, there is still a therapeutic dilemma regarding proper treatment of all types of fungal endocarditis (FE) due to the lack of controlled studies comparing the combination of antifungal and surgical therapy versus surgery or medical therapy alone. We can categorize the treatment options for fungal prosthetic endocarditis as a) antifungal monotherapy, b) combination antifungal therapy, c) surgery alone, d) surgery and antifungal, and e) surgery and combination antifungal therapy.

In a meta-analysis of surgical versus medical treatment for both prosthetic and non-prosthetic candida endocarditis, antifungal monotherapy was associated with the poorer patient outcomes compared to combination antifungal agents [20]. This observation led to the suggestion that combination antifungal therapy may be better than monotherapy for nonsurgical patients [20].

It is obviously difficult to compare clinical outcomes of medical treatment versus the combination of medications and surgery due to different baseline comorbidities and demographics. Antifungal monotherapy or sequential therapy combined with surgical approach has been used variably by different institutions [1–3, 5]. Both patients with FE and CPE tend to have high mortality rate when treated with single or multiple antifungal agents compared to those treated with combination of surgery and antifungal agents [1, 4, 10, 20, 21]. Thus current guidelines advise combined medical and surgical treatment for FE who are deemed suitable for surgery [13, 14].

However, the overall relapse which usually happens after stopping oral antifungal suppressive therapy in all types of FE can reach more than 40 % [1, 10, 21–23]. Valve tissues have been found to be positive for candida for more than two decades leading to CPE [2, 5, 24]. There is no clear recommendation in the current guidelines regarding the duration of antifungal treatment after the valve surgery but there is a trend to continue antifungal suppressive treatment for lifelong.

## Conclusions

Our patient with recurrent candida prosthetic endocarditis had a total of four valve replacement surgeries following the initial mitral annuloplasty with prolonged periods of good functionality between the surgeries. It is reasonable to argue that the recurrent surgeries along with lifelong suppressive antifungal therapy helped our patient enjoy a good quality of life with minimum hospitalization in comparison to her life on medical therapy alone.

There are not enough cases or evidence reported to establish standard guidelines for treating CPE. The current recommendations advise a combined medical and surgical approach. However we feel that based on this case it is reasonable to consider multiple redo valve replacement surgeries in conjunction with antifungal treatment as an option for selected patients stable enough to tolerate the surgery.

## Consent

Written informed consent was obtained from the patient's daughter for publication of this case report. A copy of the written consent is available for review by the Editor-in-Chief of this journal. The procedures followed were in accordance with the ethical standards of the Helsinki Declaration (1964, amended most recently in 2008) of the World Medical Association.

## Abbreviations

CPE: Candida prosthetic endocarditis
FE: Fungal endocarditis
UVa: University of Virginia
MV: Mitral valve
MVR: Mitral valve replacement
LV: Left ventricle
AVR: Aortic valve replacement
IDSA: Infectious Disease Society of America
ESCMID: European Society of Clinical Microbiology and Infectious Diseases
